# Supplementary material for: Periovulatory Subphase of the Menstrual Cycle Is Marked by a Significant Decrease in Heart Rate Variability
Source: Biology (Basel). 2023 May 29;12(6):785. doi: 10.3390/biology12060785 (PMC10295577; doi:10.3390/biology12060785)
Supplement: Supplementary file 1 [file biology-12-00785-s001.zip › biology-2283794-supplementary.pdf]

Supplementary Figure 1 : Model 1 Quantile Plot

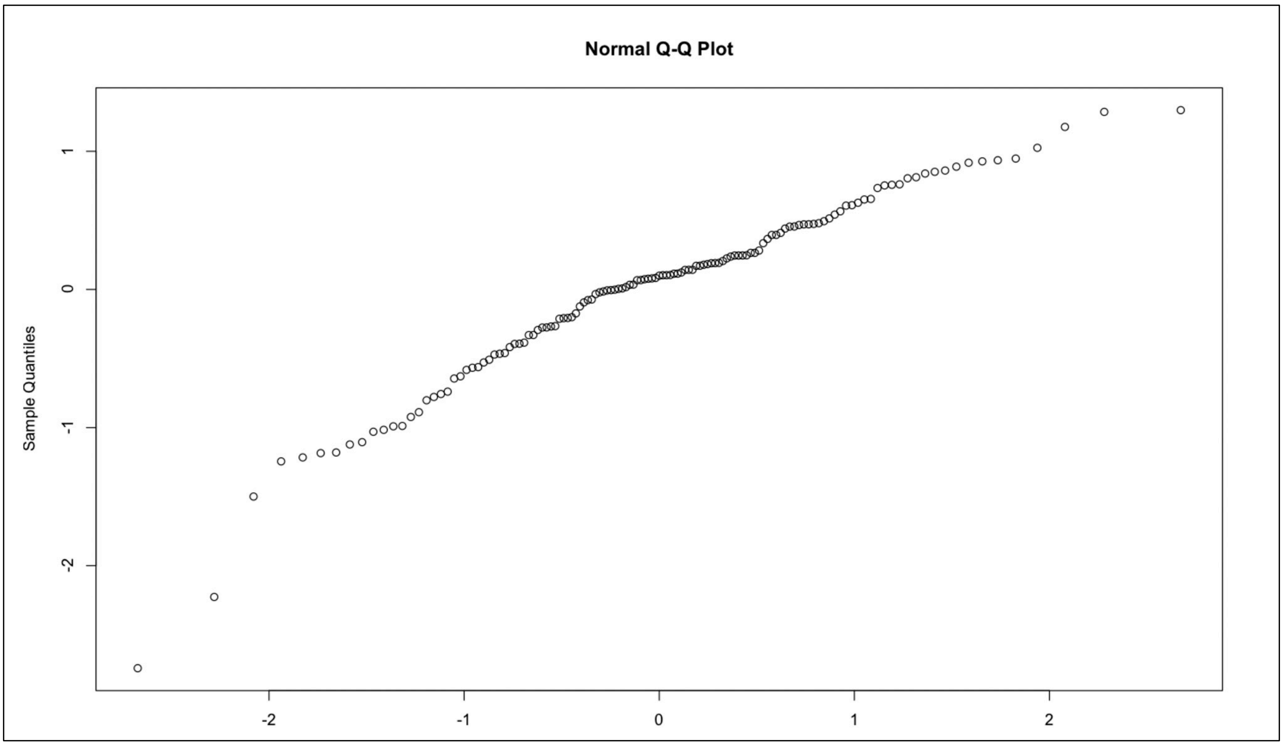

Supplementary Text

This section describes the results of analysis of heathy participants (n=16). The ICC of the unconditional means model (model 00) showed that of the total variance in HF-HRV, 70.22% was attributable to between-person variation. Including subphase as a fixed effect term in the next model (model 01), resulted in the following change from the intercept (β= 6.5380) (i.e., relative to the early follicular subphase: coded as 0): (1) periovulatory (β=-0.9949; p= 0.000797), and (2) late luteal (β=-0.7878, p=0.015220). Analysis of variance comparison showed high statistical significance between models 00 and 01 (p=0.007556). The quantile plot did not raise significant concerns regarding the normality of the weighted residuals. Pairwise comparisons of all the timepoints in model 01, with the Tukey method for comparing a family of 6 estimates, showed a statistically significant difference between early follicular and periovulatory subphases (β=0.9949; p= 0.0099).
